# Surgical Treatment of Distal Cholangiocarcinoma

**DOI:** 10.3390/curroncol29090524

**Published:** 2022-09-17

**Authors:** Leva Gorji, Eliza W. Beal

**Affiliations:** 1 Department of Surgery, Kettering Health Dayton, Dayton, OH 45405, USA; 2 Departments of Oncology and Surgery, Barbara Ann Karmanos Cancer Institute, Wayne State University School of Medicine, Detroit, MI 48201, USA

**Keywords:** distal cholangiocarincoma, hepatobiliary, malignancy

## Abstract

Distal cholangiocarcinoma (dCCA) is a rare malignancy arising from the epithelial cells of the distal biliary tract and has a poor prognosis. dCCA is often clinically silent and patients commonly present with locally advanced and/or distant disease. For patients identified with early stage, resectable disease, surgical resection with negative margins remains the only curative treatment strategy available. However, despite appropriate treatment and diligent surveillance, risk of recurrence remains high with nearly 50% of patients experiencing recurrence at 5 years subsequent to surgical resection; therefore, it is prudent to continue to optimize neoadjuvant and adjuvant therapies in order to reduce the risk of recurrence and improve overall survival. In this review, we discuss the clinical presentation, workup and surgical treatment of dCCA.

## 1. Introduction

Cholangiocarcinoma (CCA) is a rare malignancy arising from the epithelial cells of the biliary tree with an incidence of 1.3–3.4 cases per 100,000 in the western world. The malignancy is notoriously difficult to treat, as clinical symptoms often only present after patients develop locally advanced disease [1,2,3]. CCA can be classified based on its location as either intrahepatic or extrahepatic, with further subdivision of the extrahepatic category into perihilar or distal [3,4,5]. Perihilar CCA, or Klatskin tumor, is defined as arising from the right hepatic duct, left hepatic duct, or common hepatic duct; distal cholangiocarcinoma (dCCA) is specifically defined as arising beyond the junction where the cystic duct joins the common hepatic duct to form the common bile duct and proximal to the ampulla of Vater [3,4,6,7]. Approximately 30% of CCA manifest as dCCA [8].

Modifiable and non-modifiable risk factors for dCCA include choledochal cysts, primary sclerosing cholangitis (PSC), inherited mutations including Lynch Syndrome, choledocholithiasis, cholangitis, smoking, and alcoholism [9]. Surgical intervention with margin negative (R0) resection, including en-bloc removal of surrounding structures and adequate lymphadenectomy, is the only potentially curative treatment. Notably, lymph node metastasis, resection margin, tumor differentiation, and perineural invasion are the most significant prognostic indicators for 5-year survival [4,10,11,12,13]. The extent of surgical resection may vary based on the size and location of the tumor, but most resectable dCCAs require a pancreaticoduodenectomy (Whipple procedure) [13]. Segmental bile duct resection has fallen out of favor due to inadequate lymphadenectomy [14]. Ultimately, appropriate patient selection and adherence to surgical principles is imperative for successful outcomes. Patients should be carefully evaluated to ensure the absence of liver metastasis, retropancreatic and paraceliac lymph node involvement, invasion into the hepatic artery, and disseminated disease [13]. With the application of these surgical principles, 5-year overall survival (OS) is 16–66% [13,15,16]. However, recurrence remains extremely common with greater 50% of patients who underwent R0 resection demonstrating recurrent disease within 5 years; thereby, emphasizing the necessity of a collaborative and multimodal approach to treating dCCA [16,17]. In this review, we explore the presentation, workup, therapeutic strategies, and surgical treatment considerations necessary to address dCCA.

## 2. Presentation and Preoperative Considerations 

### 2.1. Presentation

Patients with dCCA typically present with nonspecific symptoms similar to other periampullary malignancies. These symptoms include painless jaundice, vague abdominal pain which may be localized to the right upper quadrant, and weight loss. Cholestatic symptoms related to obstruction including pruritus, dark or tea colored urine, clay-colored stools, and cholangitis may also be present [18,19]. Laboratory assessment often reveals elevated bilirubin, alkaline phosphatase, γ-glutamyl transpeptidase, and eventually alanine and aspartate aminotransferases [20].

### 2.2. Radiological Evaluation 

There are two important goals for the initial imaging evaluation of patients with suspected dCCA. The first is to evaluate local and regional extent of disease and the second is to evaluate for distant metastasis. The National Comprehensive Cancer Network (NCCN) provides guidelines regarding the principles of work-up and treatment for dCCA [21]. While ultrasound may have some utility in the initial diagnosis of hepatocellular carcinoma, intrahepatic cholangiocarcinoma, or hilar cholangiocarcinoma, its contribution to the diagnosis of dCCA is extremely limited [18,19]. Imaging should include a computed tomography (CT) of the chest with or without contrast for staging purposes and a contrasted multiphasic CT or magnetic resonance imaging (MRI) of the abdomen and pelvis with thin cuts to appropriately delineate the anatomy of the arterial system, portal system, and biliary tree in relationship to the tumor. While dilation of the biliary tract, lymphadenopathy, and vascular invasion can be identified with CT, this imaging method does possess limitations when evaluating intraductal tumor spread. Magnetic resonance cholangiopancreatography (MRCP) is the most accurate cross-sectional modality for identification of tumor spread within the biliary tree [18,19,21]. Of note, patients with dCCA and other periampullary neoplasms tend to have both dilated intra- and extrahepatic bile ducts and a distended gallbladder [22]. A mass lesion may or may not be identified. dCCA may result in biliary dilation alone but commonly leads to the “double duct” sign including a dilated pancreatic duct [23]. In contrast, a perihilar cholangiocarcinoma may have dilated intrahepatic ducts only with a normal caliber common bile duct and a contracted gallbladder [24].

### 2.3. Endoscopic Evlauation 

When imaging findings suggest a periampullary mass or distal bile duct stricture endoscopic evaluation is an appropriate next step. Endoscopic retrograde cholangiopancreatography (ERCP) and endoscopic ultrasound (EUS) are beneficial options for tumor inspection and biopsy. ERCP allows for direct visualization of the biliary tree, tissue sampling and biliary stent placement. ERCP biopsy and brush cytology both demonstrate excellent specificity, however, sensitivity of either modality is limited in detecting malignant biliary stricture [25,26]. EUS allows for fine-needle aspiration of biliary strictures and masses, visualization of the lymph nodes and vascular structures, while avoiding cannulation of the biliary tree [18,27].

Preoperative biliary drainage to relieve obstructive symptoms associated with dCCA may be necessary via percutaneous transhepatic biliary drainage (PTBD) or endoscopic biliary drainage (EBD). EBD is typically utilized for dCCA, as the transpapillary approach allows for better access to the tumor site. Although somewhat debated, indications for drainage include relief of obstructive jaundice, bilirubin greater than 10 mg/dL and palliative relief in patients who are not surgical candidates [28,29,30]. However, a critical complication to consider is severe cholangitis, which can occur secondary to the introduction of bacteria from the gastrointestinal tract after instrumentation of the biliary tree. Other major complications include pancreatitis, duodenal perforation, biliary perforation, tube occlusion, and bleeding [30,31]. The majority of patients with surgically resectable dCCA require pancreaticoduodenectomy. Although no significant mortality difference has been shown, increased postoperative infectious complications, wound infection, and delayed gastric emptying have been demonstrated in patients undergoing pancreaticoduodenectomy (Whipple) after preoperative biliary drainage [32]. 

In the event where relief of obstructive jaundice is required, biliary stenting options include plastic stents (PS), uncovered self-expandable metal stent (SEMS), and covered SEMS. PSs range from 5F to 12Fr in diameter and 1–18 cms in length. The ideal PS will extend 1–2 cm proximal to the lesion and 1 cm distally into the duodenum. The advantage of PSs include accessibility of the product and reduced cost [33,34]. However, studies have demonstrated that median survival is lower in patients with metastatic disease who have undergone PS placement in comparison to SEMS, 2.8 months vs. 11.6 months, respectively [35]. Furthermore, PSs have a greater rate of occlusion, which subsequently increases the risk of cholangitis and may impair the course of chemoradiotherapy in patients with resectable or borderline resectable disease [33,34,35,36]. SEMS have a characteristic immediate expansion after deployment as well as a chronic resistance to tissue compression with gradual expansion of the stent to its full capacity. Covered stents possess an external polymer coating preventing the ingrowth of tissue and allowing stent retrieval but with an associated risk of stent migration. Uncovered SEMS are nonretreivable and possess a high rate of tumor ingrowth without the increased risk of stent migration. Covered and uncovered SEMS typically range from 5 to 10.5 Fr in diameter and 4–12 cms in length, and provide comparable relief in biliary obstruction [37,38]. Classically, SEMS are recommended for patients with resectable disease [39,40]. 

### 2.4. Tumor Markers and Biologic Resectability

Carbohydrate antigen 19-9 (CA 19-9) and carcinoembryonic antigen (CEA) are tumor markers that have been utilized for evaluation of malignancies associated with the biliary tract. In addition to malignant neoplasms, these tumor markers may also be elevated in benign processes that cause inflammation or stricturing of the biliary tree [18,19,41]. CA 19-9 has also been reported as a predictor of resectability in CCA, where marked elevation of >300 KU/L is associated with unresectable disease. It can also be utilized to monitor for recurrence [42,43]. While the sensivity and specificity of the tumor markers are variable and broad, the accuracity of diagnosis is significantly improved with cytology and tissue sample [19]. Additionally, serum p53 antibodies may also aid in the early detection of dCCA, with a positive serum detection in nearly 20% of patients [44]. 

### 2.5. Multidisciplinary Evaluation – Tumor Board Discussion

Due to the poor prognosis and complexity of CCA, an interdisciplinary tumor board comprised of medical, surgical and radiation oncologists, interventional radiologists, and pathologists should be convened to optimize treatment selection and sequence. Measurable benefit for patients with CCA, and other hepato-biliary cancers, has been demonstrated with the utilization of tumor board discussion, including increased 1-, 5-, and 10-year survival [21,45,46,47,48,49].

### 2.6. Cardiac and Pulmonary Evaluation 

Patient selection requires evaluation of patients’ general health prior to surgery. Cardiovascular and pulmonary comorbidities are significant predictors of major complications [50,51,52]. Metabolic equivalents (METs) is a tool measuring the energy demand of physical activities in relation to resting metabolic rate; METs can be utilized to measuring cardiovascular functional status. Low risk surgical patients are those with ≥4 METs, and may proceed with surgery without any further cardiac testing. However, patients with a poor functional capacity, defined as <4 METs, require stress echocardiography as recommended by the American College of Cardiology (ACC) [53]. The ACC and American Heart Association (AHA) have provided preoperative guidelines of assessment for patients undergoing non-cardiac intervention. A preoperative chest x-ray is indicated in patients with known pathologies, including pneumonia or congestive heart disease. A preoperative electrocardiogram (EKG) should be undertaken in any asymptomatic male over 50 or female over 60 who has not had evaluation within the past year, or a patient of any age with diabetes, renal disease, or any type of cardiopulmonary disease [54]. In high-risk patients, cardiopulmonary exercise testing (CPET), which includes inducing symptom-limited exercise with measurements of respiratory oxygen uptake, carbon dioxide production, and ventilatory measurements, is an important tool for preoperative evaluation of high-risk patients undergoing pancreaticoduodenectomy [50,51]. Patients with a history of cardiac disease, defined as congestive heart failure, myocardial infarction, cardiac stents, and history of bypass, have a significantly higher rate of serious postoperative complication and perioperative mortality subsequent to pancreatic resection; serious complications include: pneumonia, prolonged mechanical ventilation postoperatively, stroke, abscess, MI or cardiac arrest, renal failure, sepsis, septic shock, thrombotic events, and hemorrhage with transfusion requirements [55]. Therefore, significant emphasis is placed on appropriate preoperative cardiopulmonary evaluation. Estimates of patient frailty have been introduced to allow for a more subtle evaluation of patients’ perioperative risk. For example, the Memorial Sloan Kettering – Frailty Index can be utilized to stratify perioperative risk assessment in geriatric patients over the age of 75 undergoing oncologic surgery. Each point increase in the scoring system is associated with a greater length of stay and higher likelihood of intensive care admission with a one-year mortality risk of 5% for a score of 0 and nearly 20% for scores ≥4 [56]. The preoperative assessment and optimization of health are important pillars of consideration to improve perioperative outcomes. 

### 2.7. The role of ERAS/Prehabilitation

Enhanced recovery after surgery (ERAS) is an evidence-based approach to optimizing perioperative management and outcomes of surgical patients. ERAS was first implemented in colorectal patients with the intent of reducing the surgical stress response, promoting expedited functional recovery, decreasing the duration of hospital length of stay, and reducing postoperative complications. Recommendations for the implementation of ERAS protocols specifically for patients undergoing pancreaticoduodenectomy have been developed (Table 1) [57]. Implementation of ERAS protocols for patients undergoing pancreaticoduodenectomy is associated with decreased risk of minor complications and shorter length of hospitalization; without an increase in major complications including post-operative pancreatic fistula, intraabdominal abscesses, reoperation, and mortality [58].

## 3. Prognostic Factors for Distal Cholangiocarcinoma 

Although dCCA is a rare malignancy, factors associated with prognosis have been identified (Table 2). The American Joint Committee on Cancer (AJCC) developed the eighth edition of staging for dCCA with new staging criteria (Table 3). The tumor depth of invasion is categorized as follows: T1, depth of invasion <5 mm; T2, depth of invasion between 5–12 mm; T3, depth of invasion >12 mm; and T4, tumor invasion into the celiac axis, or superior mesenterenic artery. The nodal status is classified as follows: N0, no regional lymph node metastasis; N1, regional metastasis to 1–3 lymph nodes; and N2, regional metastasis to greater than 4 lymph nodes [59]. There is a correlation between depth of invasion and other adverse pathological tumor characteristics, such as lymph node metastasis [59,60]. Specifically, T3 disease has been associated with nodal metastasis, perineural invasion, and invasion into the adjacent pancreatic tissue. T2 and T3 disease were associated with a 3-fold and 6-fold increased risk of death with a median survival of 36.5 months and 14.7 months, respectively [60]. N2 disease was associated with a significantly shorter median survival of 1.3 years versus 2.2 years in patients with fewer than 4 positive lymph nodes [10,61,62,63,64,65]. The patient’s lymph node ratio (LNR) accounts for the positive lymph node count (PLNC)/total lymph node count (TLNC), functions as an indicator for disease burden, and may be utilized as a prognostic indicator in patients with suboptimal lymph node harvest [66,67,68,69]. The value of this hybrid parameter has been demonstrated to have prognostic significance in other malignancies as well, including gastric cancer. In a meta-analysis by Kawai et al., lymph node ratio greater > 0.2 was associated with poor overall survival in patients with dCCA [64,66]. You et al. reported an even lower cutoff for LNR of > 0.10 being a poor prognostic indicator for overall survival [59]. While a formal lymph node harvest requirement has not been established by the AJCC, Kang et al. recommended that a harvest of less than 12 lymph nodes was a poor prognostic indicator of overall survival. Pancreatic invasion is also an important prognostic factor of overall survival and can be further divided into superficial (≤1 mm) and deep (>1 mm) invasion [66]. Median survival time for superficial and deep pancreatic invasion was identified to be 28 and 18 months, respectively [62,63,67]. Perineural invasion is a well-established prognostic indicator for dCCA, where 5-year OS was noted to be 32% vs. 67% in patients with versus without perineural invasion [62,63,70,71]. Finally, a microscopically negative (R0) margin is the most important prognostic factor in survival for surgically resectable tumors, as demonstrated by a 20% higher 5-year OS in comparison to incomplete margins (R1) [10,16,62,63,72]. 

## 4. Surgical Principles

### 4.1. Classic Versus Pylorus-preserving Pancreaticoduodenectomy 

Surgical resection with microscopically negative margins, including radical en-bloc resection of surrounding structures and adequate lymphadenectomy, is the only potentially curative option for dCCA. Given the location of dCCA in the common bile duct between the ampulla of Vater and the junction of the cystic and common hepatic ducts, a pancreaticoduodenectomy is typically indicated. Many studies have compared and reviewed the primary outcomes, perioperative parameters, and postoperative mortality of the classic Whipple (CW) in comparison to the pylorus-preserving pancreaticoduodenectomy (PPW) [71,72]. Previously the difference remained controversial, and studies showed mixed results regarding meaningful distinctions between the two approaches in terms of surgical outcome. Novel analysis has identified that PPW required shorter operative time and displayed lower intraoperative blood loss with associated decreased transfusion requirements; however, PPW was associated with greater risk of delayed gastric emptying (DGE) [16]. The rate of other considerations including, pancreatic fistula, bile leaks, postoperative bleeding, pulmonary complications, the necessity for re-excision, duration of hospitalization, status of resection margins, and postoperative mortality remain comparable [16,71,72]. Therefore, surgeon preference and patient-specific characteristics contribute to operative planning and the choice to proceed with CW or PPW.

### 4.2. Lymph Node Dissection

Lymph node invasion is a prognostic for overall survival in all malignancies that display lymphatic spread, however there is not a consensus regarding the number of lymph nodes that defines an adequate dissection in patients with dCCA (Table 4). The AJCC has endorsed the retrieval of at least 12 nodes for adequate lymph node staging [57,58,73,74,75]. Due to the influential impact of lymph node metastasis on treatment outcome, a study by Yoshida et al. recommended CW or PPW with extended lymphadenectomy, including periaortic lymph node dissection that extended from the celiac axis superiorly, to the inferior mesenteric artery inferiorly, the lateral margin of the inferior vena cava, and the medial margin of the abdominal aorta for optimal curative resection of dCCA. Although further studies should be pursued, limited current literature suggests a wide lymphadenectomy [12].

Skeletonization of the hepatoduodenal ligament is an important component of performing an adequate lymph node harvest for dCCA, allowing for exploration of the lymph nodes, connective tissue, autonomic nervous plexus surrounding the hepatoduodenal ligament—this includes the removal of neural plexus around the hepatic artery and portal vein. The nerve plexus located to the right side of the celiac and pancreatic head are resected; however, the SMA nerve plexus is preserved, as resection may lead to malnutrition secondary to intractable diarrhea [75].

### 4.3. Margin Negative Resection/Intraoperative Frozen Section

Achieving a negative surgical margin is critical for the surgical management of dCCA. As a legitimate potential of vascular invasion does exist, diligent operative planning is vital to achieving successful R0 resection. Intraoperative frozen sections may be utilized to assess and ensure negative margins, allowing for further excision of margins as indicated [64]. In a retrospective study by Chen et al. intraoperative frozen section was encouraged in order to ensure R0 resection, as R0 resection was associated with greater OS in comparison to R1 resection. Secondary R0 resection with intraoperative frozen section showed similar survival benefits as primary R0 resection [65]. 

### 4.4. Adjuvant Chemotherapy 

Multidisciplinary treatment of dCCA is critical and adjuvant therapy after surgical resection for dCCA is currently recommended. The BILCAP trial was a multicenter, randomized, controlled trial including 44 heptopancreatobiliary centers in the United Kingdom, which evaluated the role of adjuvant chemotherapy in patients with cholangiocarcinoma and gallbladder cancers. This trial included patients over the age of 18, whom had undergone at least R1 resection (intrahepatic CCA 19%, perihilar CCA 28%, dCCA 35%, 18% gallbladder malignancy). Patients were randomized to oral capecitabine twice daily on days 1–14 for 8 cycles based on a 21-day cycle or observation. While the primary outcome of OS based on the intention-to-treat population was not achieved, per-protocol analysis did suggest that capecitabine can improve OS when utilized as adjuvant therapy in cholangiocarincoma [79]. Other relevant trials include the PRODIGE-12 trial and the BCAT trial. The PRODIGE-12 Trial was a randomized, multicenter trial completed in France, which showed no benefit in relapse-free survival with the administration of gemcitabine and oxaliplatin (GEMOX) versus observation in patients with biliary tract malignancies [80]. The BCAT trial was a randomized, multicenter trial completed in Japan, which demonstrated no survival benefit with the use of adjuvant gemcitabine versus observation for patients with biliary tract malignancies [81]. Further investigation to optimize the role of neoadjuvant and adjuvant therapies for dCCA should be undertaken; however, eligible patients should receive adjuvant capecitabine, as it has been demonstrated to improve OS. 

## 5. Recurrence

In total, >50% of patients who undergo R0 resection experience recurrence at 5 years. NCCN guidelines recommend surveillance with imaging every 3–6 months for the first 2 years, every 6–12 months subsequently for 5 years, and on an as indicated basis [21]. Routine postoperative CA19-9 level recommendations have not yet been established, but studies have demonstrated that an elevated perioperative CA19-9 level is associated with decreased OS [82]. After curative-intent Whipple procedure for dCCA, the most common sites of locoregional recurrence are the superior mesenteric artery nodes, abdominal aortic nodes, celiac artery nodes, and nodes along the hepatoduodenal ligament [83,84,85]. The most frequent metastatic site includes the liver followed by metastasis to the peritoneum, lymph nodes, and lungs [17,86]. Given the sites of recurrence, the role of further surgical intervention is extremely limited. Due to the high risk of recurrence, diligent surveillance is extremely prudent. 

## 6. Surgical Complications after Pancreaticoduodenectomy in Patients with Distal Cholangiocarcinoma

Pancreaticoduodenectomy is the most common curative surgical intervention required for appropriate oncologic resection of dCCA. The most common complications include: pancreatic leak, delayed gastric emptying, and bile leak [17,86]. A pancreatic leak is defined as a drain amylase level greater than 3 times the upper limits of serum amylase on postoperative day 3 [87]. Drain placement subsequent to pancreaticoduodenectomy has been a well-established recommendation, as the drain output functions as an effective predictor and source of treatment for pancreatic fistula [87,88]. DGE is defined as inability to tolerate a standard diet and the requirement of prolonged nasogastric (NG) intubation [89,90]. DGE is subdivided into three different grades: grade A is defined as NG tube requirement for 4–7 days postoperatively, or re-insertion after postoperative day 3; grade B is defined as NG tube requirement for 8–14 days postoperatively, or reinsertion after postoperative day 7; grade C is defined as NG tube requirement greater than 14 days postoperatively or reinsertion after postoperative day 14 [90]. There is a greater association of DGE with pylorus-preserving pancreaticoduodenectomy in comparison with standard pancreaticoduodenectomy [89,90]. Although not as common as pancreatic fistulas, bile leak is a potential complication and can result in prolonged hospitalization. Risk factors for bile leak include male gender, decreased serum albumin levels, preoperative endoscopic biliary drainage, CBD diameter <5 mm, anastomosis of the segmental bile duct, and absence of biliary leak test [91,92,93,94,95,96]. While the most common complications of the Whipple procedure do not increase mortality, these complications do increase length of stay and may delay adjuvant therapy [97]. Patients must appropriately recover from surgery in order to be able tolerate adjuvant therapy. Early adjuvant therapy has demonstrated improved OS outcomes. Parsons et al. demonstrated a decrement in OS associated with delays in the initiation of adjuvant therapy beyond 59 days [98]. A similar principle was identified in pancreatic ductal adenocarcinoma, where delays in adjuvant therapy result in decrease in OS; however, delayed adjuvant treatment still demonstrated increased OS in comparison to surgical intervention alone [99,100].

## 7. Conclusions

Distal cholangiocarcinoma is a rare malignancy arising from the epithelial cells of the distal bile duct that is notorious for its dismal prognosis. Various prognostic indicators can be utilized in order to predict overall survival; ultimately, surgical excision with R0 resection is the only curative option in resectable disease. While appropriate candidates are treated with a pancreaticoduodenectomy, the disease process is plagued by a high rate of recurrence even in patients with margin-negative resection. Further investigation should be pursued to optimize neoadjuvant and adjuvant systemic therapy for greater overall survival and reduction in the rate of recurrence. 

## Figures and Tables

**Table 1:** ERAS recommendations for pancreaticoduodenectomy [50].

Preoperative	Intraoperative	Postoperative
Alcohol cessation: one month of abstinence.	Wound catheters/transversus abdominis plane block: conflicting results on efficacy.	PCA or IV lidocaine.
Smoking Cessation: one month of abstinence.	Avoid hypothermia: cutaneous warming.	Postoperative Nausea and Vomiting (PONV): multimodal intervention during and after surgery.
Supplements and enteral nutrition beneficial for significantly malnourished patients.	Fluid balance: avoid volume overload; fluid bolus resuscitation based on transesophageal doppler found to be beneficial. Balanced crystalloid > 0.9% NS.	Hyperglycemia should be avoided to reduce postoperative complication; however, implemented in conjunction with avoiding hypoglycemia.
Fasting: clear liquids cessation 2 h prior to surgery, solid food cessation 6 h prior to surgery with emphasis on carbohydrate intake in non-diabetics.	Perianastomotic drain: maintain for 72 h with early removal subsequently.	Transurethral advised to remove postoperative day 1 or 2.
Anti-thrombotic prophylaxis: Mechanical and chemical prophylaxis. Chemical prophylaxis with continuation 4 weeks after hospitalization. Precautions for chemical prophylaxis with the utilization of epidural.	Nasogastric tube: not preemptively indicated.	Oral nutrition in the form of small meals.
Antimicrobial prophylaxis: utilize single dose 30–60 min prior to skin incision; repeated doses as indicated based on half-life intraoperatively.		Delayed gastric emptying: artificial nutrition indicated for patients with long duration delayed gastric emptying.
Preanesthetic medication: short acting anxiolytics may be used for procedures, i.e., epidural insertion. Routine use of long-acting sedatives not advised.		Early ambulation: encouraged on morning of postoperative day 1 with daily targets.
Epidural analgesia: superior pain control with lower rates of respiratory compromised compared to IV opioids.		Stimulation of bowel: oral laxatives, chewing gum, near-zero fluid balance.

PCA—Patient-Controlled Analgesia, h—hour; IV—Intravenous; min—minute; NS—Normal Saline; PONV—Post-operative Nausea and Vomiting.

**Table 2:** Prognostic factors for distal cholangiocarcioma.

Prognostic factors	Outcomes
Depth of invasion	T2 and T3 associated with lower OS [60].
Presence of lymph node metastasis	N2 disease associated with significantly lower median survival than N1 disease [10,61,62,63].
LNR	>0.2 associated with worse overall survival [64].
Lymph Node Harvest	<12 lymph nodes harvest, associated with decreased overall survival [66].
Pancreatic invasion	Can be further categorized into ≤1 mm or >1 mm, which impact prognosis differently [62,63,67].
Perineural invasion	Indicator of poor prognosis, and decreased 5-year survival [62,63,70].
Tumor histology/differentiation	Mucin-producing vs papillary [71].
Resection Margins	Microscopically negative (R0) resection associated with more favorable OS [10,16,62,63,72].

**Table 3:** TNM staging of distal cholangiocarcinoma.

Primary Tumor (T)	Regional Lymph Nodes (N)	Distant Metastasis (M)
T1: depth of invasion <5 mm.	N0: no regional lymph node metastasis.	M0: no distant metastasis.
T2: depth of invasion between 5–12 mm.	N1: regional metastasis to 1–3 lymph nodes.	M1: distant metastasis.
T3: depth of invasion >12 mm.	N2: regional metastasis to greater than 4 lymph nodes.	
T4: tumor invasion into the celiac axis, or superior mesenterenic artery.		

**Table 4:** Selected references evaluating lymph node dissection.

Author	Study Period	N	TLNC Median	Outcomes
Kang et al. [64]	1991–2015	780	≥12	TLNC < 12 and TLNC ≥ 12 displayed significant OS difference, accounting for both node negative and node positive disease.
Kawai et al. [66]	1991–2004	62	≥12	LNR > 0.2 is an important factor predicting OS.
Kim et al. [76]	2004–2011	91	≤11	Perineural invasion prognostic indicator of OS in TLNC of ≤ 11, but not in patients with TLNC > 11.
Kiriyama et al. [61]	2001–2010	370	≥19	Median survival significantly decreased by 4+ PLNC and LNR > 0.17.
Li et al. [75]	2000–2014	448	≥12	LNR better prognostic indicator of OS than PLNC.
Lin et al. [77]	2004–2014	449	4–9	Optimal TLNC to function as prognostic indicator 4–9.
Oshiro et al. [78]	2001-–2009	60	< 12 = ≤ 12	No statistical difference between TLNC < 12 or≥12.
You et al. [59]	2002-–-2012	251	≥12	Better prediction of OS than AJCC 8th edition, using the following modified staging system, consisting of revised T category (T1: <5 mm, T2: 5–10 mm, and T3: >10 mm) and LNR ≥ 0.1.

Abbreviations: LNR, lymph node ration; PLNC, positive lymph node count; TLNC, total lymph node count.

## Abbreviations

ACC	American College of Cardiology
AJCC	American Joint Committee on Cancer
AHA	American Heart Association
CA 19-9	Carbohydrate Antigen 19-9
CCA	Cholangiocarcinoma
CEA	Carcinoembryonic Antigen
CPET	Cardiopulmonary Exercise Testing
CT	Computed Tomography
CW	Classic Whipple
dCCA	Distal cholangiocarcinoma
DGE	Delayed Gastric Emptying
EBD	Endoscopic Biliary Drainage
EKG	Electrocardiogram
ERAS	Enhanced Recovery After Surgery
ERCP	Endoscopic Retrograde Cholangiopancreatography
EUS	Endoscopic Ultrasound
h	hour
IV	Intravenous
LNR	Lymph Node Ratio
METs	Metabolic Equivalents
min	minute
MRCP	Magnetic Resonance Cholangiopancreatography
MRI	Magnetic Resonance Imaging
NCCN	National Comprehensive Cancer Network
NG	Nasogastric
NS	Normal Saline
OS	Overall Survival
PCA	Patient-controlled Analgesia
PLNC	Positive Lymph Node Count
PONV	Postoperative Nausea and Vomiting
PPW	Pylorus-preserving Pancreaticoduodenectomy
PS	Plastic Stent
PSC	Primary Sclerosing Cholangitis
PTBD	Percutaneous Transhepatic Biliary Drainage
R0	Microscopically Negative
SEMS	Self-Expandable Metal Stent
TLNC	Total Lymph Node Count
